# Molecular Abnormalities and Carcinogenesis in Barrett’s Esophagus: Implications for Cancer Treatment and Prevention

**DOI:** 10.3390/genes16030270

**Published:** 2025-02-25

**Authors:** Thaís Cabral de Melo Viana, Eric Toshiyuki Nakamura, Amanda Park, Kaique Flávio Xavier Cardoso Filardi, Rodrigo Moisés de Almeida Leite, Luiz Fernando Sposito Ribeiro Baltazar, Pedro Luiz Serrano Usón Junior, Francisco Tustumi

**Affiliations:** 1 Department of Gastroenterology, Universidade de Sao Paulo, Sao Paulo 05508-220, Brazil; 2 Department of Evidenced-Based Medicine, Centro Universitário Lusíada, Santos 11050-071, Brazil; 3 Department of Surgery, Beth Israel Deaconess Medical Center, Harvard Medical School, Boston, MA 02215, USA; 4 Department of Health Sciences, Hospital Israelita Albert Einstein, Sao Paulo 05652-900, Brazil

**Keywords:** gastroesophageal reflux, carcinogenesis, Barrett’s esophagus

## Abstract

Background: Barrett’s esophagus (BE) is described by the transformation of the normal squamous epithelium into metaplastic columnar epithelium, driven by chronic gastroesophageal reflux disease (GERD). BE is a recognized premalignant condition and the main precursor to esophageal adenocarcinoma (EAC). Understanding the molecular mechanisms underlying BE carcinogenesis is crucial for improving prevention, surveillance, and treatment strategies. Methods: This narrative review examines the molecular abnormalities associated with the progression of BE to EAC. Results: This study highlights inflammatory, genetic, epigenetic, and chromosomal alterations, emphasizing key pathways and biomarkers. BE progression follows a multistep process involving dysplasia and genetic alterations such as TP53 and CDKN2A (p16) mutations, chromosomal instability, and dysregulation of pathways like PI3K/AKT/mTOR. Epigenetic alterations, including aberrant microRNA expression or DNA methylation, further contribute to this progression. These molecular changes are stage-specific, with some alterations occurring early in BE during the transition to high-grade dysplasia or EAC. Innovations in chemoprevention, such as combining proton pump inhibitors and aspirin, and the potential of antireflux surgery to halt disease progression are promising. Incorporating molecular biomarkers into surveillance strategies and advancing precision medicine may enable earlier detection and personalized treatments. Conclusions: BE is the primary preneoplastic condition for EAC. A deeper understanding of its molecular transformation can enhance surveillance protocols, optimize the management of gastroesophageal reflux inflammation, and refine prevention and therapeutic strategies, ultimately contributing to a reduction in the global burden of EAC.

## 1. Introduction

Barrett’s esophagus (BE) is the transformation of the esophageal squamous epithelium into a metaplastic columnar epithelium [1,2]. BE has a prevalence of 1% in the Western population and increases to 5–12% in patients with gastroesophageal reflux disease (GERD), the leading risk factor [2,3]. The reflux of gastric acid into the esophagus induces cellular damage and inflammation. The body responds by creating metaplastic tissue more resistant to the hostile environment [4].

Metaplasia is the process in which one cell type substitutes another, and during this change, the cells are vulnerable to genetic changes that can result in neoplasia [4]. BE is recognized as a premalignant condition and the sole known esophageal adenocarcinoma (EAC) precursor. The progression from BE to EAC typically follows a sequence that begins with the development of dysplasia. Non-dysplastic BE progresses to EAC at an estimated rate of 0.33% annually. However, the risk increases markedly with dysplasia. BE with low-grade dysplasia progresses at approximately 1% per year, while BE with high-grade dysplasia carries a progression rate of about 6% annually [5,6].

Esophageal cancer ranks as the eighth most common cancer and the sixth leading cause of cancer-related deaths worldwide. EAC comprises only 14% of all esophageal cancers in the world but is the predominant subtype in highly developed countries [7]. The progression from BE to dysplasia and eventually to EAC comprises a series of genetic mutations, including point mutations, loss of heterozygosity, and large genomic rearrangements. Some of these genetic changes are consistent across various stages of carcinogenesis, while others are more stage-specific, occurring at distinct points during the neoplastic progression [8,9].

Identifying molecular abnormalities and pathways involved in the BE–dysplasia–neoplasia sequence is crucial for improving therapeutic targeting and determining prognosis. This study aims to review these molecular abnormalities in BE, laying the groundwork for developing personalized strategies in the future.

## 2. Methods

This is a narrative review of the molecular abnormalities associated with the carcinogenesis of Barrett’s esophagus.

### 2.1. Database Search

A non-systemic review search was performed in Embase, Cochrane, PubMed, and LILACS, as well as a manual search of references. The search covered the inception of the databases to September 2024. The following search terms were used: “molecular abnormalities”, “epigenetic alterations”, “carcinogenesis”, “Barrett esophagus”, and “esophageal adenocarcinoma”.

### 2.2. Study Selection

The inclusion criteria were as follows: (a) studies that evaluated patients with a histologically confirmed diagnosis of Barrett’s esophagus; (b) studies that evaluated cancer molecular panels and/or genetic abnormalities; (c) studies that evaluated the carcinogenesis of BE and/or progression to EAC; and (d) Portuguese or English articles.

The exclusion criteria were as follows: (a) reviews, case reports, editorials, letters, and abstracts; and (b) no full-text availability.

Two reviewers (T.C.M.V. and F.T.) searched and selected the studies based on the previously defined eligibility criteria.

### 2.3. Data Extraction and Main Outcomes

Relevant data were extracted from the selected studies by two researchers (T.C.M.V. and F.T.). Key information included the study’s characteristics (author, year, and study design), the molecular abnormalities investigated (e.g., genetic mutations, epigenetic changes, and chromosomal alterations), and the outcomes related to BE progression to dysplasia or cancer. Additional details on the clinical implications of these findings were also collected.

### 2.4. Data Synthesis

A qualitative synthesis was conducted to summarize and interpret the findings from the reviewed literature. This approach allowed for the integration of diverse data sources, including different study designs, to construct a comprehensive narrative on the molecular abnormalities and carcinogenesis in Barrett’s esophagus.

## 3. Results

Following the study selection process, a total of 160 studies were included in this non-systematic review. These studies encompassed a diverse range of research designs, including pre-clinical investigations that explored molecular mechanisms and pathways underlying BE progression to dysplasia or cancer. This review also incorporated findings from observational studies and clinical trials at various stages, offering insights into potential therapeutic targets and interventions aimed at preventing or managing EAC.

Although there is no single mutation or distinct cluster of mutations that exclusively differentiates Barrett’s metaplasia from EAC, the progression from BE to cancer is generally viewed as a cumulative process involving multiple mutations and molecular changes. This accumulation ultimately disrupts the regulatory mechanisms controlling cellular proliferation and apoptosis.

In the following sections, we outline the key molecular alterations, categorizing them into inflammatory, genetic, epigenetic, and chromosomal alterations.

### 3.1. Chronic Inflammation

**Figure 1:**
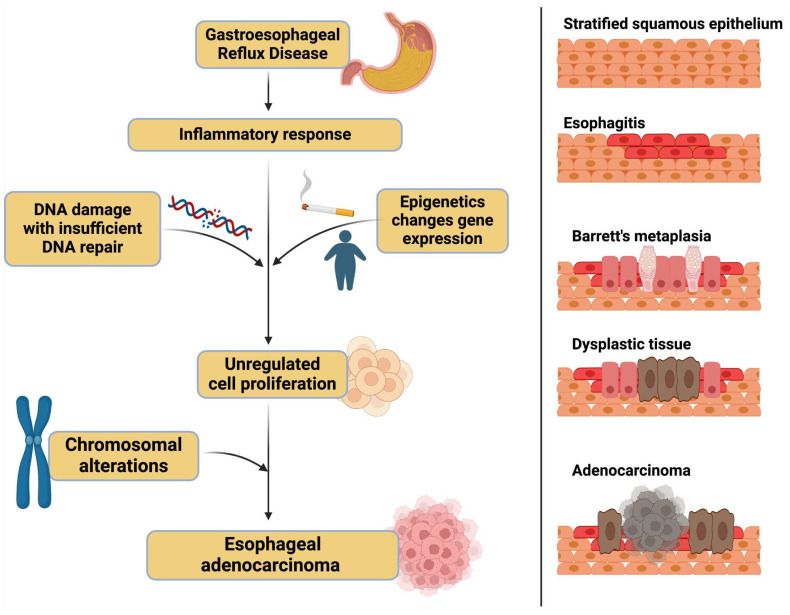
A didactic example of the progression from gastroesophageal reflux disease (GERD) to esophageal adenocarcinoma. This figure highlights the different processes—inflammatory, genetic, epigenetic, and chromosomal alterations. It is important to note that these processes are dynamic and concurrent and do not necessarily follow this exact sequence.

Chronic inflammation is a key driver in the development of various malignancies and is estimated to contribute to nearly 25% of human cancers [10]. It facilitates tumorigenesis through diverse mechanisms, including the induction of DNA damage, promotion of angiogenesis, and disruption of critical cellular processes such as growth, proliferation, and programmed cell death [11]. These inflammatory processes establish a microenvironment that not only supports malignant transformation but also promotes tumor progression and resistance to therapeutic interventions. See Figure 1.

Since *Helicobacter pylori* was discovered by Warren and Marshall in 1982 [12], its strong link to gastric cancer has been well established, with over 95% of gastric cancer patients testing positive for infection [13]. The chronic inflammation from reflux esophagitis [14] also significantly increases the esophageal and esophagogastric cancer risk. Exposure to bile salts and acid can lead to chronic inflammation and esophageal injury [15]. Under typical conditions, inflammation of the esophageal squamous epithelium resolves through the regeneration of new squamous cells. However, in certain instances, the healing process leads to the replacement of these cells with Barrett’s cells instead [16]. Once BE develops, the newly formed intestinal-type columnar cells exhibit greater resistance to the harmful agents responsible for chronic inflammation, unlike the native esophageal squamous tissue [17].

**Figure 2:**
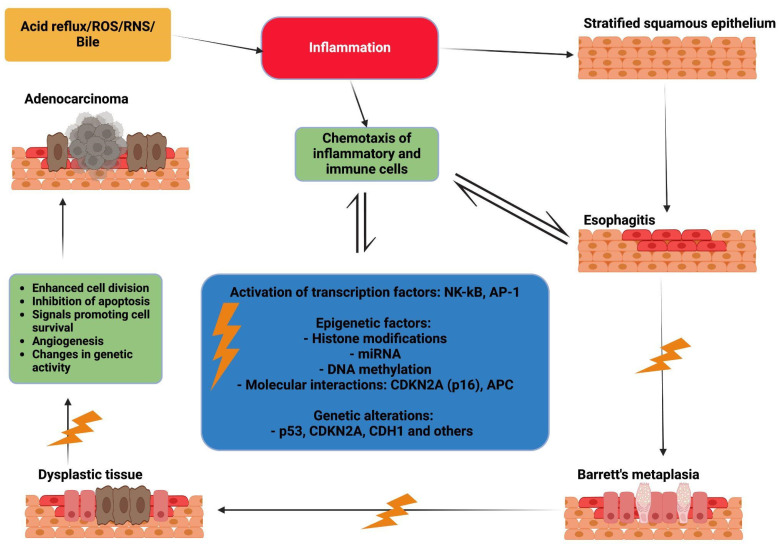
Proposed mechanism of inflammation in the progression from metaplasia to dysplasia and adenocarcinoma in the esophagus. Reactive oxygen species (ROS) and reactive nitrogen species (RNS).

This prolonged inflammatory state triggers an increased release of pro-inflammatory molecules, including prostaglandins, cytokines, chemokines, and reactive oxygen (ROS) or nitrogen species (RNS) [18]. The immune, mesenchymal, and epithelial cells produce various mediators that not only regulate inflammation but also contribute to its persistence and eventual progression to cancer. Among these mediators, cytokines such as interleukin (IL)-1β, tumor necrosis factor (TNF)-α, and IL-6 play key roles in establishing the link between chronic inflammation and carcinogenesis [19]. See Figure 2.

Interleukin (IL)-1β and TNF-α exert direct effects on epithelial cells by triggering the activation of the nuclear factor-κB (NF-κB) transcription factor [20,21]. This activation plays a pivotal role in both the inflammatory response and the progression to cancer. Although NF-κB and inflammation are primarily associated with innate immunity—a defining feature of multicellular organisms—its impact extends beyond immune function. Innate immunity is one of six core principles essential to metazoan life, which also include controlled cell replication and growth, programmed cell death, cell–cell and cell–matrix adhesion, regulated developmental processes, and cell type specialization [22]. Through its activation, NF-κB contributes to the regulation of these fundamental biological processes.

NF-κB contributes to regulated cell replication and growth by inducing proliferative genes, regulates programmed cell death by controlling genes encoding anti-apoptotic molecules [23], and promotes the induction of proliferative genes [24,25]. Additionally, NF-κB promotes the production of growth factors and cytokines, including epidermal growth factor (EGF) and interleukin-6 (IL-6). It also enhances the induction of cyclooxygenase-2 (COX-2) and amplifies the generation of reactive oxygen species (ROS) [26]. The induced cyclooxygenase-2 (COX-2) plays multiple roles, including promoting cell growth and angiogenesis [27]. These cytokines concurrently activate mitogen-activated protein kinase (MAPK) cascades, which are integral to various carcinogenic processes. For instance, TNF-α and IL-6 activate the extracellular signal-regulated kinase (ERK)/MAPK pathway, a critical signaling cascade involved in cell proliferation, migration, and angiogenesis [28].

TNF-α activates the IκB kinase (IKK) [29] signaling cascade via the TNF receptor 1 (TNFR1) [29] and the TRADD/receptor-interacting protein 1 (RIP1)/TNF receptor-associated factor 2 (TRAF2) complex [29,30], ultimately resulting in the activation of NF-κB transcription factors. While immune cells are widely recognized as the primary source of TNF-α, autocrine production by cancer cells also plays a significant role, particularly under conditions where NF-κB is constitutively activated within the tumor microenvironment [31,32]. Notably, elevated TNF-α and NF-κB expressions have been reported in both BE and esophageal adenocarcinoma (EAC), suggesting their involvement in the progression from BE to malignancy [33].

Interleukin-6 (IL-6) is a versatile cytokine essential for immune regulation and hematopoiesis [34]. Beyond its normal physiological roles, IL-6 has been strongly linked to cancer development and progression [34]. It is produced not only by immune cells but also by various other cell types, including epithelial cells and osteoblasts [35]. A particularly noteworthy aspect of IL-6 is its production by cancer cells, suggesting an autocrine role in tumor growth. By stimulating anti-apoptotic and angiogenic pathways, IL-6 contributes to tumor advancement [36]. Supporting this idea, Wang et al. demonstrated increased IL-6 mRNA expression in paraffin-embedded tissue samples using in situ hybridization, along with elevated serum IL-6 levels detected via ELISA in patients with squamous cell carcinoma in the esophagus [37]. Dvořáková et al. in 2024 [38] observed in a cohort of 15 known Barrett’s esophagus (BE) patients that elevated IL-6 levels in BE tissue may contribute to the development of apoptosis resistance. This resistance to programmed cell death could increase the susceptibility of the affected epithelium to malignant transformation.

Moreover, IL-6 triggers the activation of the signal transducer and activator of transcription 3 (STAT3), which plays a crucial role in promoting cell proliferation. This activation also drives the synthesis of growth factors, including the Regenerating Gene (Reg) protein [39]. The Reg protein has been implicated in the development of gastrointestinal inflammation and its progression to cancer [40,41,42,43]. The constitutive activation of signal transducer and activator of transcription 3 (STAT3) has been identified as a significant contributor to oncogenesis, with activated STAT proteins observed across various human cancers [44,45]. The STAT family comprises STAT1, STAT2, STAT3, STAT4, STAT5A, STAT5B, and STAT6 [46], each of which has distinct biological functions. These proteins regulate key processes such as cell survival, differentiation, metabolism, and immune responses and are critically involved in the development of malignant tumors and autoimmune diseases [47]. Notably, tumor necrosis factor-α (TNF-α) and IL-6 frequently establish a positive feedback loop that drives cancer progression.

The interplay between these cytokines and STAT3 signaling establishes a feedback loop that perpetuates inflammation and epithelial damage while promoting a microenvironment conducive to metaplasia and malignant transformation. STAT3 signaling represents a key intrinsic pathway in cancer-related inflammation, as it is often activated in malignant cells and drives the expression of numerous genes essential for promoting inflammatory processes [48].

Chronic inflammation also plays a key role in driving the transformation of normal cells into cancerous ones capable of clonal expansion. Research has shown that oxidative DNA damage is present in Barrett’s mucosa, often exceeding the cell’s ability to repair it [49]. As a result, individuals with Barrett’s esophagus face a significantly higher risk—ranging from 30 to 125 times—of developing esophageal adenocarcinoma (EAC) compared to the general population [50].

During inflammation, both inflammatory and epithelial cells generate ROS and RNS, which contribute to widespread damage affecting nucleic acids, proteins, and lipids [51]. These reactive molecules can cause DNA lesions, leading to mutations such as 8-oxo-7,8-dihydro-2′-deoxyguanosine (8-oxodG) and 8-nitroguanine, which are associated with carcinogenesis [52,53,54]. Notably, the formation of 8-nitroguanine is an important marker of oxidative DNA damage and it is considered a pivotal molecular event that is commonly associated with various types of inflammation-related carcinogenesis.

Chronic exposure to elevated levels of reactive oxygen species (ROS), caused by persistent reflux and impaired bolus clearance in the esophagus, results in excessive free radical activity that attacks genomic DNA. This damage can lead to various types of DNA lesions, including single-strand and double-strand breaks, through the increase in (8-OH-dG) and p-H2AX, which in turn contribute to genomic instability [55]. Elevated levels of 8-OH-dG formation were observed on the apical surface of columnar epithelial cells in biopsy specimens from patients with BE and BE-associated EAC, suggesting that oxidative and nitrative DNA damage plays a significant role in the carcinogenesis associated with BE [56].

Oxidative stress in BE leads to DNA strand breaks, which activate nuclear enzymes known as poly(ADP-ribose) polymerases (PARPs) [57]. These enzymes facilitate the process of poly(ADP-ribosyl)ation (PARylation) at sites of DNA damage. Using NAD+ as a substrate, PARPs covalently attach negatively charged ADP-ribose (ADPr) groups to target proteins, with arginine, aspartic acid, and glutamic acid residues being the primary attachment sites [55]. Once the initial ADPr is added, additional ADPr units can be polymerized, forming linear or branched poly(ADP-ribose) (PAR) structures [58]. Among the PARP family, PARP-1 is the most abundant and well-studied member.

Excessive oxidative DNA damage can overwhelm PARP-1’s repair capacity, leading to its overactivation. This results in significant consumption of NAD+, which requires ATP for its synthesis. Consequently, cellular NAD+ and ATP depletion can disrupt metabolism, ultimately leading to cell death [58]. Thus, while PARP-1 is crucial for maintaining genomic integrity, its excessive activation can have detrimental effects, making it a double-edged sword in DNA damage response mechanisms [55].

Although research on PARP-1’s role in BE development and GERD-related esophageal cancer is limited, preliminary findings suggest that PARP-1 overexpression may confer resistance to oxidative stress and bile acid-induced damage in BE epithelial cells [59]. Additionally, PARP-1 appears to enhance the survival of esophageal epithelial cells, positioning it as a potential therapeutic target in BE [59].

Beyond its role in DNA repair, PARP-1 is also a co-activator of NF-κB, a transcription factor central to inflammatory processes [60,61]. PARylation of NF-κB by PARP-1 promotes its activation, amplifying inflammation [62]. In PARP-deficient models, NF-κB activity is significantly impaired, while PARP-1 inhibition in inflammatory conditions like contact hypersensitivity reduces oxidative stress and NF-κB activation, thereby alleviating inflammation [63].

In the context of BE, PARP-1 may act as a negative regulator of p63, a protein associated with epithelial cell identity, by enhancing NF-κB activity. This suggests that oxidative stress-induced upregulation of PARP-1 could suppress p63 expression in BE-related stem cells, potentially contributing to the pathogenesis of BE and its progression to malignancy [55].

Chronic inflammation and recurrent pH changes in the esophageal mucosa due to GERD lead to heat shock protein (HSP) expression changes [64,65]. HSPs are pivotal in modulating immune responses by interacting with pro-inflammatory and anti-inflammatory pathways. HSPs are a family of highly conserved molecular chaperones that play a critical role in maintaining cellular homeostasis, particularly under stress conditions such as heat, oxidative stress, and inflammation. Their primary function is to assist in properly folding proteins, prevent protein aggregation, and facilitate the repair or degradation of damaged proteins, being crucial to apoptosis regulation [66]. Heat shock proteins (HSPs) may play a protective role during the early stages of cancer initiation, such as in chronic esophagitis, but their expression patterns change as cancer progresses [67].

Research utilizing an animal model of reflux esophagitis demonstrated that the expression of HSP 27 mRNA is significantly elevated in the distal esophagus of rats with esophagitis compared to controls [68]. In contrast, HSP 70 expression diminishes following thermal injury to the esophageal epithelium but recovers and increases during the healing phase of esophagitis [69]. This suggests a dynamic role for heat shock proteins (HSPs) in the esophageal response to injury and subsequent repair [70].

Numerous studies have underscored the protective role of Hsp27 protein expression in mitigating a broad spectrum of cytotoxic stresses [71,72,73,74,75,76]. Its high abundance in the esophageal stratified squamous epithelium suggests that Hsp27 functions as a critical cellular defense mechanism, safeguarding the esophagus from diverse cytotoxic insults and contributing to tissue resilience [77].

Another factor supporting the protective role of Hsp27 is the observed 60% higher rate of neoplastic progression of Barrett’s in men compared to women [78]. While the biological basis for this gender disparity remains unclear, one possible explanation is that women possess more effective protective mechanisms in the esophagus that mitigate damage and reduce the risk of metaplasia and neoplasia.

Hsp27 expression is regulated by estrogen in certain breast carcinoma cell lines [79] and is also observed in estrogen-responsive organs such as the female reproductive tract (oviduct, vagina, and uterus) [80]. Furthermore, studies have shown that Hsp27 mRNA expression is 4.1-fold higher in women (*p* < 0.02) [77]. These findings suggest a potential link between Hsp27 levels and estrogen’s protective effects, which may contribute to the reduced susceptibility of women to Barrett’s metaplasia and esophageal neoplasia.

The regulation of HSP 27 (encoded by Hspb1) in esophageal endothelial cells exposed to acidic environments is governed by the phosphatidylinositol 3-kinase (PI3K) and p38 mitogen-activated protein kinase (MAPK) pathways [81]. Acidic conditions stimulate the phosphorylation of PI3K and MAPKs, which catalyzes the phosphorylation of HSP 27 [81,82]. This post-translational modification facilitates a structural remodeling of HSP 27, breaking large oligomers into smaller functional units [83]. These smaller units play a critical role in cellular defense mechanisms.

HSP 27 interacts with Akt (protein kinase B) to inhibit the release of cytochrome c (Cyt C) from mitochondria, a pivotal step in the apoptosis cascade [83]. Additionally, HSP 27 regulates apoptosis by modulating the activity of apoptosis signal-regulating kinase 1 (Ask1), an MAPK family member, and by influencing the function of the Fas receptor (CD95), a key death receptor on the cell surface [83]. Another protective mechanism of HSP 27 includes reducing the accumulation of reactive oxygen species (ROS), thereby mitigating oxidative damage and further preventing apoptosis [84].

Through these pathways, HSP 27 not only shields cells from injury but also promotes esophageal epithelial cell proliferation. This protection, however, comes with potential risks, as the deregulation of apoptosis is a well-established step in carcinogenesis. Thus, while HSP 27 is integral to cell survival and repair, its dysregulation could contribute to the progression from injury to malignancy.

While HSP 27 plays a critical role in esophageal injury and repair, findings by Zhang et al. suggest its expression remains unchanged in patients with esophagitis, regardless of whether Barrett’s esophagus is present [85]. However, their study revealed significant molecular differences between esophagitis with and without Barrett’s esophagus. Patients with Barrett’s esophagus exhibited notably lower levels of HSP 70 and HSP 90α compared to those with esophagitis alone [85]. Additionally, telomerase reverse transcriptase (TERT) expression was reduced in Barrett’s esophagus, while the expression of HSP 105 and Caspase-3 was increased [85].

The reduction in TERT expression, a key regulator of telomerase activity, highlights a potential vulnerability in Barrett’s epithelium to DNA instability due to shortened telomeres [85]. This instability is exacerbated by the increased expression of Caspase-3, a critical executor of apoptosis, suggesting an environment predisposed to cellular turnover and genomic disruptions [64]. The diminished levels of HSP 70 and HSP 90α, which are crucial for counteracting oxidative stress, may further contribute to the persistence and chronicity of the preneoplastic state in Barrett’s esophagus [85].

Conversely, the elevated expression of HSP 105 in Barrett’s epithelium may serve as a compensatory cytoprotective mechanism, mitigating the effects of inflammation and oxidative damage [64]. HSP 105 has been shown in previous studies to reduce lipid peroxidation and maintain the integrity of the mucosal barrier under stress conditions [84,86]. This duality underscores the complex interplay between protective and pathological mechanisms within Barrett’s epithelium.

The heterogeneity in cellular proliferation and apoptosis along the length of Barrett’s epithelium suggests that the progression to cancer is not uniform but rather driven by a network of interconnected carcinogenic pathways. The imbalance in protective versus damaging factors creates a microenvironment primed for neoplastic evolution, highlighting the need for targeted interventions to disrupt these pathways. These findings offer valuable insights into the molecular underpinnings of Barrett’s esophagus and its progression to esophageal adenocarcinoma.

### 3.2. Genetic Alteration

Different chromosomal alterations can lead to the gain of function of proto-oncogenes or the loss of function of tumor suppressor genes. Proto-oncogenes can drive cell proliferation through various mutations: chromosomal translocation, in which a gene is relocated from a transcriptionally inactive site to a transcriptionally active one; point mutation, where the substitution of a single base leads to an amino acid change in the oncoprotein; gene amplification, resulting in the incorporation of multiple copies of an oncogene; and the insertion of a promoter gene near a proto-oncogene, leading to its overexpression. Conversely, tumor suppressor genes are essential for regulating normal cell growth and differentiation. For a cancer cell to achieve unchecked proliferation, both copies of a tumor suppressor gene must be rendered inactive. One mechanism contributing to this inactivation is the loss of heterozygosity, which can result from chromosomal deletions, mitotic errors, or intermediate genetic deficiencies. This process is believed to signify the loss of crucial tumor suppressor genes, thereby promoting cancer development [87].

The development of EAC from BE is marked by the accumulation of genetic mutations that drive the malignant transformation of esophageal epithelial cells [88]. These genetic changes disrupt key regulatory pathways, promoting uncontrolled cell growth, resistance to apoptosis, and genomic instability, which are hallmarks of cancer progression.

In inflammatory conditions, cellular levels of ROS increase significantly, leading to the generation of modified nucleic acid bases, such as oxidatively altered pyrimidines and purines [89]. These modifications can result in various forms of DNA damage, including single- and double-strand breaks, DNA intra-strand adducts, and DNA–protein cross-links [90]. Additionally, ROS impairs mismatch repair mechanisms, allowing the accumulation of mutations in microsatellite sequences [91].

Oncogene activation contributes to genomic instability in both premalignant conditions and cancer cells. In this context, ROS serves as a mediator linking the excessive activity of oncogenes to DNA damage [92,93]. These observations suggest that chronic ROS production, a condition known as oxidative stress, plays a crucial role in the initiation and progression of inflammation-associated cancers by driving genetic instability.

#### 3.2.1. Point Mutations

Point mutations represent one of the most prevalent genetic alterations in EAC. These mutations involve the substitution of a single nucleotide in the DNA sequence, which can significantly impact gene function. A notable example is the mutation of the TP53 tumor suppressor gene, frequently detected in Barrett’s esophagus (BE). TP53 encodes the p53 protein, a key regulator of the cell cycle and DNA repair. When mutated, p53 loses its ability to halt the cell cycle in response to DNA damage, resulting in genomic instability and fostering tumor development. Additionally, the loss of functional p53 disrupts apoptosis, enabling damaged cells to evade programmed cell death and continue proliferating [2,94].

The relationship between chromosome 17p, specifically the TP53 gene, and BE is well established in the medical literature. TP53 encodes the p53 protein, a critical regulator of the DNA damage response and cell cycle arrest. Mutations in TP53 impair the ability of p53 to maintain genomic integrity, leading to defective DNA repair and uncontrolled cell proliferation. Alterations in TP53, such as mutations, are early and common events in the progression of BE to EAC. The dysfunctional p53 protein not only fails to suppress tumor formation but may also gain oncogenic functions, further driving the progression of BE toward malignancy [95].

In BE and EAC, p53 gene alterations are commonly observed, similar to other types of cancer. These alterations often involve the loss of gene function through allelic loss or mutations. Loss of heterozygosity (LOH) at the p53 locus is detected in 75–80% of EAC cases, 79% of high-grade dysplasia (HGD), 42% of low-grade dysplasia (LGD), and 14% of Barrett’s metaplastic tissue. This indicates that LOH at the p53 locus is an early and significant change in the progression from BE to EAC [96,97,98]. In a retrospective cohort by Stachler et al., TP53 mutations were identified in 46% of patients who progressed to EAC, compared to only 5% of those who did not (*p* < 0.0001; OR 13.8; 95% CI = 3.2–60.5) [99].

p53 mutations are commonly found in both BE and EAC, affecting 40–88% of cancers and 30–66% of Barrett’s tissues with mild or no dysplasia. Among these mutations in Barrett’s tissue, 50–80% are missense mutations, which involve GC to AT transitions at CpG islands, while 10–50% are nonsense mutations. In HGD and EAC, mutations are typically located between codons 152 and 306, with the most frequent changes occurring at specific CpG dinucleotide hotspot codons (175, 196, 213, 245, 248, 273, and 282). These mutations interfere with p53’s ability to bind DNA and regulate stress response genes, leading to the accumulation of genetic errors. This accumulation is passed on to daughter cells, promoting cancer progression [100,101]. Indirect activation of p53 also occurs because of mdm2 overexpression, a known regulator of p53 activity. Soslow et al. showed that in cases without p53 mutation, overexpression of mdm-2 occurred in 50% of cases and might be responsible for the loss of tumor suppressor [102] function.

Ireland et al. investigated the clinical implications of p53 mutations and found that patients harboring these mutations tended to be younger than those without them. Additionally, their study revealed that individuals with p53 mutations developed more aggressive tumors and had significantly poorer prognoses [103]. In a separate prospective study, Schneider et al. [104] examined the prognostic impact of p53 mutations in patients who underwent curative resection for EAC arising from BE. Their findings indicated that p53 mutations were present in 50.8% of cases and had a substantial effect on survival outcomes. Specifically, the 5-year survival rate was 68% in patients without the mutation, compared to only 24% in those with p53-mutated tumors. Based on these results, p53 mutational status was identified as a key high-risk factor for treatment failure following curative surgery.

According to the retrospective cohort by Redston et al., the influence of TP53 mutations on progression to EAC appears to be independent of the presence or absence of dysplasia in baseline biopsies. The odds ratio for progression was 58 (95% CI = 17.9–188.5; *p* < 0.0001) in patients without dysplasia, 49.5 (95% CI = 10.0–245.0; *p* < 0.0001) in patients with indefinite dysplasia, and 17.8 (95% CI = 6.4–49.5; *p* < 0.0001) in those with low-grade dysplasia [105].

Furthermore, TP53 alterations are more frequent and occur earlier in patients who progress to EAC compared to the classic morphological features of dysplasia used in current diagnostic practice. This opens new possibilities for the early identification of high-risk patients and treatment stratification based on this risk [105].

However, due to the low sensitivity and specificity reported in studies and the lack of robust, high-quality evidence, the use of TP53 as a standalone diagnostic tool is not currently recommended. Instead, it may serve as a complementary method for risk stratification alongside traditional histological analysis [2].

#### 3.2.2. Deletion

Another significant genetic alteration in BE is the loss-of-function mutations or deletions in CDKN2A (p16). This gene encodes a cyclin-dependent kinase inhibitor that regulates the G1/S checkpoint of the cell cycle. When CDKN2A is inactivated, the cell cycle progresses uncontrollably, leading to excessive cell proliferation. This deregulation is a key step in the transition from normal epithelial cells to dysplastic or neoplastic cells, facilitating the progression toward EAC [106].

Located on chromosome 9p21, the CDKN2A/p16 gene plays a significant role in the neoplastic progression of BE. As previously mentioned, gene alterations are common in precursor lesions and esophageal adenocarcinomas linked to BE. The inactivation of p16 can occur through various mechanisms, including somatic mutations, loss of heterozygosity (LOH), and promoter hypermethylation, with the latter being one of the most common modes of inactivation [106].

Yan-Song et al. investigated the molecular mechanisms driving progression in esophagectomy samples, focusing on CDKN2A promoter hypermethylation, LOH, and p16 expression. Promoter hypermethylation was found in 82% of adenocarcinomas and 30% of premalignant lesions, including 33% with intestinal metaplasia, indicating that hypermethylation is an early event in neoplastic transformation (*p* = 0.0002). LOH at the CDKN2A locus was observed in 68% of adenocarcinomas and 55% of premalignant lesions. Protein expression of p16, assessed by immunohistochemistry, was absent in 86% of adenocarcinomas and 27% of premalignant lesions (*p* < 0.0001) [106].

Galipeau et al. examined the sequence of genetic events in patients with simultaneous LOH at 9p (location of p16) and 17p. In 52% of cases, LOH at 9p preceded LOH at 17p, while the inverse was observed in only 5%. These findings suggest that 9p alterations are often early events in neoplastic progression. Clonal expansion was observed at different levels, with diploid clones harboring 9p LOH demonstrating greater dissemination potential along Barrett’s segment compared to clones with LOH at 17p or both loci (*p* = 0.05). These findings highlight that LOH at 9p and 17p, particularly 9p, plays a central role in early neoplastic progression, preceding events such as aneuploidy [107]. Similarly, fluorescence in situ hybridization mapping by Brankley et al. demonstrated that CDKN2A loss (9p21) was the earliest and most frequent alteration, observed in 47% of areas with intestinal metaplasia (IM) and significantly more common in IM than in areas with dysplasia or adenocarcinoma (*p* < 0.0001). Polysomy was the dominant alteration in dysplasia and adenocarcinoma, detected in 57% of low-grade dysplasia, 88% of high-grade dysplasia, and 100% of adenocarcinomas (*p* < 0.0001) [108].

Genetic analysis also aids in risk stratification for patients with BE. A cohort study by Sepulveda et al. utilized high-resolution analyses to characterize somatic copy number alterations (SCNAs) in non-dysplastic tissues from BE patients before the development of dysplasia or adenocarcinoma. Sixteen progressors (pre-progression) were compared with forty-two non-progressors. Frequent alterations in progressors included deletions involving FHIT (69%) and CDKN2A/B (69%), which were significantly more common than in non-progressors (5–14% and 19–24%, respectively). Combined SCNAs demonstrated a specificity of 76% (95% CI: 65 to 99%) and a sensitivity of 88% (95% CI: 49 to 90%) for distinguishing progressors from non-progressors. Reduced p16 expression was observed in metaplastic intestinal epithelium from progressors (*p* = 0.023), corroborating genomic findings [109].

Genetic and epigenetic changes in CDKN2A/p16 are pivotal for understanding BE progression to esophageal adenocarcinoma. These insights could guide future therapeutic strategies and improve risk stratification for patients.

#### 3.2.3. Amplifications

Gene amplifications are another form of genetic alteration that can drive tumorigenesis. In BE, amplifications of genes such as EGFR (epidermal growth factor receptor) and MYC have been identified. The amplification of EGFR leads to overexpression of the receptor, which activates downstream signaling pathways, promoting resistance to apoptosis, cell survival, and proliferation. This contributes to the uncontrolled growth characteristic of malignant tumors [110].

The MYC proto-oncogene is notably recognized as a key driver in the progression of various malignancies. In esophageal adenocarcinoma (EAC), its influence is also evident in the BE-EAC sequence, being regulated by the Ephrin B2 Receptor Tyrosine Kinase (EphB2). EphB2, a receptor tyrosine kinase, plays a central role in this process, exhibiting context-specific functions across different cancers. Unlike other gastrointestinal tumors, such as colorectal cancer, where EphB2 expression is lost during the adenoma-to-adenocarcinoma transformation, its activation persists in BE-EAC, promoting tumor progression [111].

EphB2 acts as a positive regulator of MYC, which is associated with the transdifferentiation of squamous epithelium (SQ) into a BE-like columnar phenotype. Additionally, factors such as bile acid reflux induce MYC expression, while EphB2 serves as an upstream regulator, stabilizing MYC through its interaction with MYCBP2, an E3 ubiquitin ligase. This interaction prevents MYC degradation via ubiquitination, underscoring EphB2’s critical role in maintaining the cellular plasticity associated with BE and supporting the differentiation of progenitor cells into BE-like epithelium. The presence of EphB2 and MYC in esophageal submucosal glands (ESMGs), potential progenitor sources for BE, further supports the hypothesis that activation of this axis precedes the development of BE [111].

In the prospective cohort by Rygiel et al., involving 99 patients initially diagnosed with BE, MYC expression was directly associated with the progression from BE to EAC. This alteration was most frequently observed in cases of high-grade dysplasia and EAC, further reinforcing its role in disease advancement [110].

In conclusion, EphB2, and consequently the MYC gene, plays a critical role in the development and progression of BE-EAC by connecting MYC stability to essential molecular cascades. These findings open new opportunities for therapeutic interventions and preventive strategies in this highly aggressive and prevalent malignancy.

The EGFR gene (Epidermal Growth Factor Receptor) plays a central role in the progression from Barrett’s esophagus to esophageal adenocarcinoma and has become a focus of increasing interest in molecular studies due to its clinical relevance. EGFR encodes a transmembrane protein that, when activated by its ligands, triggers intracellular signaling cascades regulating processes such as cell growth, proliferation, survival, and differentiation.

In the same prospective cohort cited above by Rygiel et al., 99 patients were also analyzed to assess the relationship between EGFR and the BE-EAC sequence. The results indicated that genetic gains in the analyzed loci are detectable even at early stages, such as in cases of BE without dysplasia, but occur at significantly higher frequencies in more advanced stages, including high-grade dysplasia and esophageal adenocarcinoma. EGFR amplifications were observed in 19% of adenocarcinoma cases, while less pronounced gains of this locus were already detectable in early stages, suggesting that these alterations may precede more severe amplification events [110]. Pretto et al. investigated the expression of EGFR in esophageal biopsies from 194 patients divided into three groups: gastroesophageal reflux disease (GERD), BE, and EAC. Immunohistochemical results showed EGFR positivity in 8.7% of GERD patients (11/127), 25% of BE patients (6/24), and 46.5% of adenocarcinoma patients (20/43), with statistically significant differences among the groups (*p* = 0.0001). These findings suggest a progressive increase in EGFR expression as disease severity advances, highlighting its potential role in the transition from benign to malignant esophageal conditions [112].

Not just a marker of progression, EGFR may also serve as a future tool for early disease diagnosis. Cronin et al. conducted an analysis of over 100 samples at different stages of progression and demonstrated a progressive and significant increase in EGFR expression along the metaplasia–dysplasia–adenocarcinoma sequence, with overexpression detected in 35% of high-grade dysplasia (HGD) cases and 80% of adenocarcinomas. Furthermore, EGFR expression was higher in BE tissues near areas of HGD and adenocarcinoma, suggesting its role in neoplastic transformation. As a membrane protein, EGFR not only reflects histological progression but also serves as a useful target to guide endoscopic biopsies, enhancing its clinical relevance in managing BE and detecting malignancies at early stages [113].

It is worth noting that some studies have not demonstrated a clear correlation between EGFR expression and the progression of reflux esophagitis, BE, or EAC, suggesting that this relationship is complex and likely influenced by multiple factors. In a case–control study, Deissova et al. examined the association between polymorphisms in the epidermal growth factor (EGF) gene and its receptor (EGFR), along with their mRNA expression and EGF plasma levels, in relation to the development of esophagitis, BE, and EAC. Their study, conducted in a Central European population, included 301 patients diagnosed with RE, BE, or EAC, as well as 106 control participants.

The results did not find a significant association between genetic polymorphisms or the EGF-EGFR genotypic interaction and these conditions (*p* > 0.05). Furthermore, EGF and EGFR mRNA expression did not vary significantly between esophageal tissues with or without visible pathological changes or among the different diagnoses (*p* > 0.05), suggesting that these biomarkers are not determinants of the risk or progression of BE and EAC [114].

### 3.3. Epigenetic Changes

Epigenetic alterations involve modifications in gene regulation that do not change the DNA sequence itself [115]. These changes are essential for controlling gene expression and cellular function, serving as a crucial link between environmental influences and genetic activity. In Barrett’s esophagus (BE), epigenetic modifications are thought to occur before genetic mutations, creating conditions that favor neoplastic progression. These alterations impact fundamental cellular mechanisms, including proliferation, apoptosis, and differentiation, promoting the transformation from normal esophageal epithelium to metaplastic and eventually dysplastic states.

Collectively, these epigenetic alterations not only contribute to the pathogenesis of BE but also highlight potential therapeutic targets. By modulating these reversible modifications, interventions aimed at restoring normal gene expression profiles may help prevent the progression from BE to esophageal adenocarcinoma, offering promising avenues for treatment and disease management.

#### 3.3.1. Histone Modifications 

Histones [116] are proteins that organize DNA into chromatin and regulate gene expression. Acetylation relaxes chromatin, promoting transcription, while deacetylation compacts it, silencing genes. Methylation can either activate or repress transcription, depending on the site. Histone modifications, such as acetylation, methylation, and ubiquitination, represent chemical alterations primarily targeting lysine and arginine residues on histones—most notably H3 and H4, but also H2A and H2B [117]. These modifications are critical in shaping the epigenetic landscape of Barrett’s esophagus (BE), directly influencing gene expression and chromatin structure. Among these modifications, epigenetic silencing in mammalian cells is mediated by at least two distinct histone modifications: polycomb-based histone H3 lysine 27 trimethylation (H3K27TriM) and H3K9 dimethylation [118]. Cancer cells exploit these silencing mechanisms by employing DNA hypermethylation to suppress critical functional pathways, facilitating tumor progression [119].

Additionally, aberrant gene silencing can also occur through mechanisms involving histone modifications, such as the induction of H3K27me3 [120]. Aberrant H3K27me3 has been observed in the promoter regions of 200–600 genes, although the majority are also likely to be passenger changes without functional roles in cancer [120].

#### 3.3.2. MiRNA

MicroRNAs (miRNAs) are non-coding RNAs, typically 20 to 22 nucleotides in length, excised from precursor RNA structures ranging from 60 to 110 nucleotides. These miRNAs are involved in various biological processes, such as development, differentiation, apoptosis, and cell proliferation, by binding to messenger RNAs (mRNAs), which carry the genetic code for proteins. Consequently, they play a central role in both transcriptional and post-transcriptional regulation and can function as either tumor suppressors or oncogenes (oncomir) [121].

Non-coding RNAs also contribute to epigenetic regulation in Barrett’s esophagus (BE). Dysregulated expression of miRNAs, such as miR-21 and miR-192, has been implicated in modulating key pathways like apoptosis and the epithelial-to-mesenchymal transition (EMT), a critical process for invasion and metastasis. These miRNAs serve as post-transcriptional regulators, fine-tuning the expression of numerous genes. Similarly, long non-coding RNAs (lncRNAs) have emerged as important regulators of chromatin remodeling, transcription, and RNA stability. Their dysregulation in BE promotes oncogenic signaling and creates a favorable environment for cancer development. See Table 1.

#### 3.3.3. DNA Methylation

DNA methylation is one of the most extensively studied epigenetic mechanisms in Barrett’s esophagus (BE). In cancer cells, both regional hypermethylation and global hypomethylation have been observed [122,123]. Regional hypermethylation refers to the abnormal DNA methylation of promoter CpG islands, which are typically unmethylated under normal physiological conditions [124,125]. When methylation occurs aberrantly in a promoter CpG island, it consistently leads to the silencing of the downstream gene [126]. In BE, hypermethylation of tumor suppressor gene promoters, such as CDKN2A (p16) and APC, silences these crucial genes. Loss of their function disrupts cell cycle regulation and apoptosis, thereby creating an environment conducive to cellular transformation and disease progression. These methylation patterns are often detectable in the early stages of disease, suggesting their potential as biomarkers for early detection and risk stratification. On the other hand, global hypomethylation contributes to carcinogenesis by promoting genomic instability [127].

DNA methylation typically occurs on a cytosine in a CpG dinucleotide. Regions dense in CpG sites are known as CpG islands and are frequently located in the regulatory regions of genes, such as promoters [128]. Research by Alvarez et al. has identified specific CG dinucleotide loci that are targeted by differential methylation during neoplastic progression, and these loci are located outside traditional CpG islands. Recent studies have similarly shown that cytosines outside CpG islands can be aberrantly methylated or hypomethylated in cancer [129].

Aging has been identified as an initial inducer of aberrant DNA methylation, followed by chronic inflammation, as evidenced by the aberrant methylation of specific tumor suppressor genes in noncancerous colonic mucosae of patients with inflammatory bowel disease (IBD) [130,131]. Such methylation changes are particularly prominent in cancers associated with chronic inflammation, including Barrett’s esophagus (BE), further highlighting the role of the inflammatory microenvironment in promoting epigenetic alterations linked to carcinogenesis [132].

DNA hypermethylation, leading to the loss or reduced expression of genes involved in cell cycle regulation, DNA repair, growth factor signaling, metastasis, and apoptosis, is a hallmark of the transformation to BE and its progression to dysplasia and adenocarcinoma [133]. Smith et al. [134] analyzed the methylation status of several genes—APC, CDKN2A, ID4, MGMT, RBP1, RUNX3, SFRP1, TIMP3, and TMEFF2—in esophageal adenocarcinoma (EAC), high-grade dysplastic BE, and metaplastic BE from patients without dysplasia or adenocarcinoma, as well as in histologically normal esophageal squamous epithelium. Their results revealed an increased frequency of methylation in EAC compared to squamous epithelium for all nine genes. Notably, while seven genes showed no significant difference in methylation frequency between BE and EAC, the methylation of CDKN2A and RUNX3 was significantly higher in EAC. Furthermore, the extent of methylation was significantly greater in EAC compared to BE for six genes (CDKN2A, ID4, RBP1, RUNX3, SFRP1, and TMEFF2), suggesting that methylation of these genes occurs early in the progression of BE, with increased methylation as the disease advances toward cancer.

Schulmann et al. [135] highlighted that hypermethylation of p16, RUNX3, and HPP1 occurs early in BE-associated neoplastic progression, serving as predictive markers for progression risk. Jin et al. identified five genes (NELL1, TAC1, SST, AKAP12, and CDH13) frequently methylated early during BE-associated neoplastic progression [136,137,138,139,140]. Their retrospective, multicenter validation study led to the development of a methylation-based risk stratification model, which accurately predicted high-grade dysplasia (HGD) and EAC cases that would otherwise have been missed, demonstrating the potential for these markers in clinical practice [141].

Moinova et al. previously reported that de novo methylation of the vimentin gene (mVIM) is a highly sensitive biomarker for BE, detectable in approximately 90% of BE patient biopsies, suggesting its potential as a screening tool [142]. They also demonstrated the feasibility of non-endoscopic molecular cytology screening for BE and EAC. By bisulfite sequencing, they identified CCNA1 methylation as another marker for BE. Their molecular cytology assay, based on a two-marker panel of mVIM and CCNA1, detected BE and EAC with over 90% sensitivity and specificity, showing the utility of DNA methylation markers in esophageal cancer surveillance [143]. Similarly, Yu et al., using a four-marker panel, achieved 80% sensitivity for HGD and EAC, with 96.3% specificity for non-dysplastic BE and normal squamous tissues, further supporting the potential of these markers for clinical use [144].

Alvi et al. focused on methylation of imprinted and X-chromosome genes, identifying four genes (SLC22A18, PIGR, GJA12, and RIN2) that best distinguished between BE and dysplasia/EAC in a retrospective cohort, with an area under the curve of 0.988. Their methylation panel successfully stratified patients into low-, intermediate-, and high-risk groups based on the number of methylated genes, providing a tool for detecting dysplasia and early-stage cancer missed by conventional histopathology [145]. Kawakami et al. found that the high plasma levels of hypermethylated APC DNA were associated with reduced survival, highlighting its potential as a prognostic biomarker for EAC [146].

In contrast, Alvarez et al. [129] reported that global cytosine hypomethylation is a more common epigenetic alteration during BE progression. Hypomethylation occurs early in multistep carcinogenesis, even in the first discernible metaplastic lesions of the squamous esophagus. It is proposed that many aberrant promoter CpG island methylation events are passenger changes, not directly contributing to carcinogenesis [147].

Jammula et al. identified four distinct subtypes of BE/EAC, each characterized by unique patterns of DNA methylation, mutation, and gene expression. Subtype 1 displayed DNA hypermethylation, a high mutation burden, and mutations in cell cycle and receptor tyrosine signaling genes. Subtype 2 showed gene expression linked to metabolic processes such as ATP synthesis and fatty acid oxidation, with a lack of methylation at certain transcription factor binding sites; 83% of samples in this subtype were BE, and 17% were EAC. The third subtype exhibited no significant methylation changes compared to control tissue but showed immune cell infiltration and had the shortest survival times. Subtype 4, characterized by DNA hypomethylation, was associated with structural rearrangements and copy number alterations, particularly amplification of CCNE1, a gene that may indicate sensitivity to CDK2 inhibitors [148].

#### 3.3.4. Main Molecular Interactions

Methylation of the promoter regions of certain genes, as described, is associated with the development of BE. This occurs because methylation can suppress the transcription of genes that would normally have an anti-tumoral effect. One potential advantage of using gene methylation as a biomarker is that its presence or absence can be quickly determined with a single PCR. Several well-established genetic alterations have served as starting points for a better understanding and the creation of predictive models for the progression from BE to EAC. Among the various known mutations, CDKN2A and APC are some of the most studied.

##### CDKN2A (p16)

**Figure 3:**
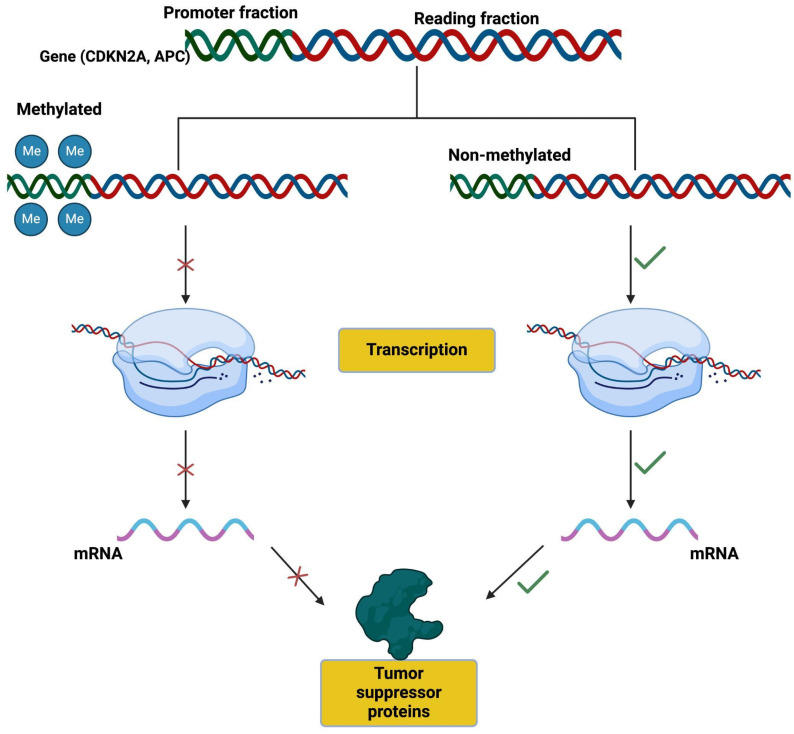
Promoter methylation suppresses gene transcription.

The p16 gene is an inhibitor of cyclin-dependent kinases and a tumor suppressor involved in various solid tumors and lymphomas. See Figure 3.

The mechanisms of p16 inactivation are diverse, including loss of heterozygosity, point mutations, and/or methylation of the p16 gene promoter. In EAC, homozygous deletion of the p16 gene and point mutations in the remaining allele of p16 are infrequent. Hypermethylation of the p16 gene promoter region is considered one of the most common genetic alterations in the progression from BE to dysplasia and EAC [149].

Fu et al. [150] demonstrated that acid, a major component of refluxate in patients with BE, increases ROS production in Barrett’s mucosal biopsies. This increase is blocked by the NADPH oxidase inhibitor apocynin, suggesting that NADPH oxidases mediate the acid-induced rise in H_2_O_2_ production. Jie Hong et al. [149] found that exogenous H_2_O_2_ significantly increased promoter methylation of the p16 gene and cell proliferation in BAR-T and OE33 cells. These findings suggest that ROS may mediate acid-induced p16 hypermethylation and enhance cell proliferation.

Wang et al. [151] showed that patients with BE who progressed from baseline pathology to high-grade dysplasia (HGD) or adenocarcinoma had a significantly higher prevalence of hypermethylation in their initial esophageal biopsies compared to those who did not progress, for both p16 (100% vs. 33%; *p* = 0.008) and APC (86% vs. 40%; *p* = 0.02). Chueca et al. [152], in a more recent study, analyzed 77 esophageal biopsy specimens from BE and/or EAC patients collected between 2000 and 2010 to assess methylation status. Their results revealed increasing rates of p16 methylation as histological lesions progressed. However, they could not establish a threshold value to predict a higher risk of developing esophageal adenocarcinoma. Sepulveda et al. [109] found that CDKN2A copy number alterations occur at a significantly higher frequency in non-dysplastic BE of patients who progress to dysplasia or cancer. This suggests that these changes may play a key role in the early stages of progression or serve as biomarkers for other mechanisms driving early progression.

##### APC

The hypermethylation of the APC gene promoter region and its consequent inactivation have been studied in various neoplasms, including Barrett’s esophagus. It is known that APC is a negative regulator of the Wnt/β-catenin pathway, and the nuclear accumulation of β-catenin is associated with tumorigenesis.

Kawakami et al. [146] demonstrated in their study that hypermethylation of the APC gene promoter region was present in abnormal esophageal tissue from 48 (92%) of 52 patients with esophageal adenocarcinoma, 16 (50%) of 32 patients with esophageal squamous cell carcinoma, and 17 (39.5%) of 43 patients with Barrett’s metaplasia. However, this hypermethylation was not found in matching normal esophageal tissues.

In a systematic review by Wang et al. [153], the main finding was a significant association between APC promoter methylation and an increased risk of Barrett’s esophagus (BE) and esophageal cancer (EC). Specifically, APC methylation was 23 times more likely to predict EC and 10 times more likely to predict BE, although the data came from heterogeneous sources. APC promoter methylation appears to be an ideal cancer biomarker, as a previous study showed it to be an early event in various malignancies. However, Wang et al. were unable to evaluate the role of APC methylation in the progression of esophageal cancer.

In a study conducted in the Japanese population [154], only APC gene hypermethylation was identified as an independent predictive marker for the development of EAC (OR = 24.4, 95% CI 2.14–277.8, *p* = 0.01), among biomarkers such as microsatellite instability, methylation status of the APC, CDKN2A, hMLH1, RUNX3, and MGMT genes, immunoreactivity of the monoclonal antibody Das-1 for the colonic phenotype, and Ki-67 staining.

#### 3.3.5. Other Hypermethylated Genes in Barrett’s Carcinoma

As described above, understanding hypermethylated [116] genes in BE has significant clinical applications, particularly in risk stratification, early detection, and prognosis of esophageal adenocarcinoma. Epigenetic alterations, such as promoter hypermethylation of tumor suppressor genes (e.g., APC, TIMP3, CDH13), can serve as biomarkers to identify patients at higher risk of progression from BE to cancer. Additionally, these markers can improve endoscopic surveillance strategies and potentially guide targeted epigenetic therapies, offering a more personalized approach to managing BE and preventing malignant transformation.

This epigenetic alteration facilitates tumorigenesis by inactivating key protective mechanisms, promoting uncontrolled cell growth, and enhancing the risk of malignant transformation in BE. Table 2 summarizes the genes associated with hypermethylation. The loss of function of these genes may contribute to the progression of BE to EAC, and ongoing research has focused on this issue [128].

### 3.4. Chromosomal Alterations

Chromosomal alterations play a significant role in the progression from BE to adenocarcinoma. These changes occur early in the course of the disease and may serve as biomarkers for the risk of cancer progression. These alterations encompass a spectrum ranging from focal chromosomal gains or losses to the identification of tetraploidy.

Aneuploidy, a chromosomal alteration characterized by an abnormal number of chromosomes in a cell, is closely associated with cancer progression in BE. Similarly, tetraploidy, defined as cellular populations with 4N DNA content, plays a pivotal role in neoplastic progression. Tetraploidy is an early marker of genetic instability and is associated with progression to aneuploidy, a more advanced stage of chromosomal instability [155].

In a prospective analysis of over 320 patients, Reid et al. demonstrated that the presence of tetraploidy or aneuploidy in initial biopsies significantly increased the relative risk of progression to EAC, with a relative risk of 19 (95% CI = 4.7, 78; *p* < 0.001) over five years, even in patients with or without low-grade dysplasia [156]. In the prospective cohort study by Chao et al., which included 362 participants, high levels of tetraploid fractions were strongly associated with an increased risk of progression from BE to EAC (*p* < 0.0001). Another notable finding of this study was the correlation between high tetraploid fractions and the presence of TP53 mutations, which, as previously discussed, are strongly associated with BE and EAC [157].

Moreover, the identification of tetraploidy appears to be a critical marker of genomic instability and is associated with rapid BE-to-cancer progression, potentially preceding this diagnosis by up to 24 months [158].

### 3.5. The Combination of Genetic, Epigenetic, and Chromosomal Alterations in Predictive Models

Several studies have attempted to develop predictive models for the risk of BE progressing to dysplasia and EAC, using a combination of genetic, epigenetic, and chromosomal markers along with clinical factors. Although no model has been universally established in clinical practice, these studies highlight the growing importance of molecular alterations in understanding BE.

The study by S.J.M. Hoefnagel et al. [159] focused on creating a predictive model to identify patients with BE who are at higher risk of developing dysplasia and EAC by analyzing chromosomal and genetic alterations. They used fluorescence in situ hybridization (FISH) on brush cytology specimens to assess abnormalities on chromosomes 7 and 17, as well as structural abnormalities in genes such as c-MYC, CDKN2A, TP53, Her-2/neu, and 20q. They combined these genetic biomarkers with clinical variables to develop a predictive model after an extended follow-up period with a median time of 7 years. Results showed a sensitivity of 0.91 and a specificity of 0.38. The positive predictive value was 0.13 (95% CI 0.09 to 0.19), and the negative predictive value was 0.97 (95% CI 0.93 to 0.99). These data indicate that while the model is highly sensitive, its specificity is relatively low, suggesting a considerable number of false positives.

Di Pietro et al. [160] aimed to improve the diagnosis of dysplasia in Barrett’s esophagus (BE) by combining chromosomal, genetic, and epigenetic alterations to identify the most significant biomarkers. Using a combination of autofluorescence imaging (AFI)-guided biopsies and molecular markers, they concluded that aneuploidy, p53 immunohistochemistry, and cyclin A were the biomarkers most strongly associated with high-grade dysplasia (HGD) and early cancer (EC). This led to the proposal of a three-biomarker panel, which demonstrated high diagnostic accuracy (AUC = 0.97) and reduced the number of required biopsies by 4.5 times compared to standard surveillance protocols. The findings suggest a more objective, efficient, and targeted approach to identifying dysplasia in BE.

Another predictive model [161] was developed in 2015 by Margriet R. Timmer et al., also aiming to improve the accuracy of identifying BE patients at higher risk of progression to dysplasia and EAC. The study developed a predictive model for BE progression using only non-dysplastic BE patients. Six molecular markers were evaluated: p16, p53, Her-2/neu, 20q, MYC, and aneusomy. Univariate analysis identified p16 loss, MYC gain, and aneusomy as the strongest predictors. The final model, integrating age, Barrett’s segment length, and an “Abnormal Marker Count” (counting abnormalities in p16, MYC, and aneusomy), significantly improved risk stratification. Patients with a higher marker count had an 8.7-fold increased risk of progression, with an AUC of 0.76. This model offers a potential tool for personalized surveillance strategies in BE.

## 4. The Future of Research on Carcinogenesis in Barrett’s Esophagus: Prevention, Surveillance, and Precision Medicine

Understanding the molecular underpinnings of carcinogenesis in BE is essential for shaping the future of clinical research and patient care. As the primary precursor to EAC, BE demands a deeper investigation into the genetic, epigenetic, and cellular mechanisms driving its progression. These insights can serve as a cornerstone for future clinical research, advancing strategies in prevention, surveillance, and precision medicine.

### 4.1. Prevention Strategies: The Role of Chemoprevention and Surgery

Chemoprevention emerges as a promising area in preventing carcinogenesis in BE. The randomized AspECT trial demonstrated that a combination of high-dose proton pump inhibitors (PPIs) and aspirin significantly delayed the development of EAC and high-grade dysplasia in BE patients. However, the aspirin dose used in the study (300 mg/day) exceeds the standard dose for cardiovascular prevention (75 mg/day), potentially increasing the risk of gastrointestinal bleeding [162].

Antireflux surgery, such as laparoscopic total fundoplication, can be more effective in reducing the risk of progression to cancer. A prospective longitudinal study with a mean follow-up of 80 months found fundoplication superior to omeprazole in controlling GERD symptoms, reversing metaplastic epithelium, and preventing dysplasia and cancer development. While surgery stops reflux entirely, medical therapy primarily controls acidity and cannot remove anatomical anomalies, such as hiatal hernia [163].

However, both chemoprevention and antireflux surgery lack standardized protocols for BE. Choosing the appropriate therapeutic approach requires considering individual factors, such as BE extent, GERD symptoms, risk of complications, and patient preferences.

### 4.2. Individualized Surveillance: Considering Risk Factors

Enhancing surveillance strategies for BE requires a shift from generalized protocols to a more individualized approach grounded in a comprehensive understanding of molecular pathways and biomarkers. While endoscopic surveillance remains vital for identifying dysplasia and EAC at an early stage, current guidelines predominantly focus on the grade of dysplasia, overlooking other key risk factors such as smoking status, age, gender, ethnicity, and the length of BE segments [164].

By integrating molecular biomarkers into surveillance protocols, clinicians can better stratify patients based on their personalized risk of progression to EAC. Advances in molecular biology have revealed numerous genetic and epigenetic alterations associated with BE carcinogenesis, including mutations in TP53, dysregulated microRNAs, and changes in DNA methylation patterns. These molecular insights could be leveraged to identify patients at higher risk, enabling more targeted surveillance schedules and interventions.

Combining molecular markers with clinical and demographic data could lead to a precision-based surveillance framework, optimizing the timing and frequency of endoscopic evaluations. By focusing on the interplay between molecular changes and clinical risk factors, the future of BE surveillance can become more personalized, effective, and aligned with the principles of precision medicine.

### 4.3. Precision Medicine: Identifying Molecular Targets

Precision medicine has the potential to revolutionize BE management by identifying specific molecular pathways involved in carcinogenesis and personalizing prevention and treatment strategies.

A study by Zaninotto et al. [165] demonstrated that fundoplication was more effective than medical therapy in reversing intestinal metaplasia and reducing the transcription factor Cdx2 expression, implicated in BE formation. This finding suggests that surgical intervention can modulate molecular pathways relevant to disease progression.

Neureiter et al. [166] compared the molecular profiles of endoscopic submucosal dissection (ESD) specimens with those of surgically resected EAC specimens. The study revealed that ESD-resected specimens had higher levels of epithelial protein biomarkers, while surgically resected tissues showed increased expression of mesenchymal proteins. These findings suggest that molecular biomarkers may differ between the two groups, and in the future, they could potentially play a role in guiding the decision between endoscopic or surgical resection.

Future research should focus on identifying molecular biomarkers predictive of EAC progression, discovering novel therapeutic targets, and developing chemoprevention strategies targeting specific molecular pathways. Precision medicine can help identify patients at higher risk of progression, enabling the implementation of personalized interventions that maximize treatment efficacy while minimizing side effects.

### 4.4. Artificial Intelligence (AI)

AI has emerged as a valuable tool to assist pathologists in diagnosing and monitoring BE and its progression to EAC. A key challenge in BE surveillance is the subjectivity involved in histopathological assessments, which can result in variability when grading dysplasia. AI-driven deep learning models, trained on whole-slide images (WSIs), have shown high accuracy in detecting dysplasia, offering pathologists a consistent and reliable second opinion. In a recent study [167], an AI model successfully classified BE-related dysplasia in 76.4% of cases, outperforming the diagnostic accuracy of most participating pathologists. Furthermore, AI-assisted computer-aided endoscopic surveillance can improve lesion detection, enhancing the identification of subtle dysplastic changes that might otherwise go undetected.

### 4.5. The Role of PPIs and Nutrition

Proton pump inhibitors (PPIs) are commonly used to manage GERD and BE, effectively reducing stomach acid and protecting the esophageal lining from damage. While these medications offer significant benefits, long-term use can have unintended consequences, such as reduced absorption of vitamin B12 and iron and alterations in gut microbiota, which may impact overall health [168]. Despite these risks, nutritional assessment is often overlooked, with only 26% of oncology centers including nutrition specialists in multidisciplinary teams. Given the increasing recognition of nutrition’s role in disease progression and treatment outcomes, it is essential to incorporate routine nutritional screening for patients with BE and EAC. Addressing malnutrition and micronutrient deficiencies early can improve treatment tolerance, recovery, and overall quality of life. By including nutrition specialists in BE surveillance programs, healthcare teams can provide more comprehensive care, ensuring that patients receive not just medical treatment but also the necessary dietary support to maintain their health.

## 5. Conclusions

BE is the primary preneoplastic condition associated with the development of gastroesophageal adenocarcinoma. Understanding the molecular abnormalities and pathways driving carcinogenesis in BE can refine surveillance strategies to identify high-risk patients more accurately and detect malignancy at an earlier, more treatable stage. Furthermore, insights into these molecular transformations can guide the development of targeted prevention strategies, including chemoprevention and personalized therapeutic interventions. Advancing our knowledge in this area holds the promise of transforming BE management, ultimately reducing the burden of gastroesophageal adenocarcinoma.

## Figures and Tables

**Table 1:** The table highlights some examples of miRNAs that appear to play a role in the oncogenesis of Barrett’s esophagus [116].

miRNA	Function in Cell	Role in Barrett’s Esophagus
**miR-192**	Tumor suppressor inhibits cell proliferation and induces apoptosis	Downregulated in BE; reduced expression is linked to higher progression risk
**miR-194**	Tumor suppressor or oncomir	Upregulated in BE and EAC; associated with metaplasia and neoplastic progression
**miR-215**	Tumor suppressor or oncomir; regulates cell differentiation and apoptosis	Upregulated in BE but downregulated in EAC; potential biomarker for progression
**miR-203**	Tumor suppressor regulates cellular growth and differentiation	Downregulated during progression from normal esophagus to EAC
**miR-205**	Tumor suppressor; involved in epithelial–mesenchymal transition (EMT)	Lower expression in BE and EAC compared to normal tissue; regulates EMT

**Table 2:** Several genes have been studied in Barrett’s esophagus and esophageal adenocarcinoma. The hypermethylation of these genes has been associated with their loss of function, the development of dysplasia and cancer, or even the expansion of Barrett’s epithelium.

Gene	Protein	Effect on Cell
AKAP12	A-kinase anchoring protein 12	Regulates β2-adrenergic receptor signaling, involved in cell adhesion
GPS &GST	Glutathione-S-transferase superfamily (GST) and glutathione peroxidase (GPX)	Cellular antioxidants
CDH13	Cadherin-13 (H-cadherin/T-cadherin)	Tumor suppressor regulates cell–cell adhesion
DAPK1	Death-associated protein kinase 1	Induces apoptosis, tumor suppressor
TAC1	Tachykinin precursor 1	Involved in neuropeptide signaling, anti-apoptotic effects
TIMP3	Tissue inhibitor of metalloproteinases-3	Inhibits tumor growth, angiogenesis, and promotes apoptosis
WIF1	Wnt inhibitory factor 1	Inhibits Wnt signaling, tumor suppressor
SFRP1	Secreted frizzled-related protein 1	Inhibits Wnt signaling, tumor suppressor
SFRP2	Secreted frizzled-related protein 2	Inhibits Wnt signaling, tumor suppressor
SFRP4	Secreted frizzled-related protein 4	Inhibits Wnt signaling, tumor suppressor
MGMT	O-methylguanine-DNA methyltransferase	DNA repair enzyme
NELL1	Protein kinase C binding protein	Control cell differentiation and growth
SOCS	STAT-induced inhibitors	Cytokine-inducible negative regulators of cytokine signaling
SST	Somatostatin	Regulates exocrine and endocrine secretion and motor activity. It is the primary inhibitor of gastrin-stimulated gastric acid secretion

## Data Availability

No new data were created or analyzed in this study. Data sharing is not applicable to this article.
